# The Chemokine CCL2 Promotes Excitatory Synaptic Transmission in Hippocampal Neurons via GluA1 Subunit Trafficking

**DOI:** 10.1007/s12264-024-01236-9

**Published:** 2024-07-02

**Authors:** En Ji, Yuanyuan Zhang, Zhiqiang Li, Lai Wei, Zhaofa Wu, Yulong Li, Xiang Yu, Tian-Jia Song

**Affiliations:** 1 Institute of Neuroscience, CAS Center for Excellence in Brain Science and Intelligence Technology, Chinese Academy of Sciences, Shanghai, 200031 China; 2 University of Chinese Academy of Sciences, Beijing, 100049 China; 3 State Key Laboratory of Membrane Biology, School of Life Sciences, Peking-Tsinghua Center for Life Sciences, and IDG/McGovern Institute for Brain Research, Peking University, Beijing, 100871 China; 4 Institute of Genetics and Developmental Biology, Chinese Academy of Sciences, Beijing, 100101 China; 5 Shandong Provincial Key Medical and Health Laboratory of Psychiatric Genetics of Shandong Mental Health Center, Shandong University, Jinan, 250014 China

**Keywords:** Synaptic transmission, CCL2, MCP-1, CCR2, CaMKII, AMPA receptor, GluA1

## Abstract

The CC chemokine ligand 2 (CCL2, also known as MCP-1) and its cognate receptor CCR2 have well-characterized roles in chemotaxis. CCL2 has been previously shown to promote excitatory synaptic transmission and neuronal excitability. However, the detailed molecular mechanism underlying this process remains largely unclear. In cultured hippocampal neurons, CCL2 application rapidly upregulated surface expression of GluA1, in a CCR2-dependent manner, assayed using SEP-GluA1 live imaging, surface GluA1 antibody staining, and electrophysiology. Using pharmacology and reporter assays, we further showed that CCL2 upregulated surface GluA1 expression primarily via Gα_q_- and CaMKII-dependent signaling. Consistently, using i.p. injection of lipopolysaccharide to induce neuroinflammation, we found upregulated phosphorylation of S831 and S845 sites on AMPA receptor subunit GluA1 in the hippocampus, an effect blocked in *Ccr2*^*−/−*^ mice. Together, these results provide a mechanism through which CCL2, and other secreted molecules that signal through G-protein coupled receptors, can directly regulate synaptic transmission.

**Supplementary Information:**

The online version contains supplementary material available at 10.1007/s12264-024-01236-9.

## Introduction

Cytokines are small secreted proteins with well-characterized functions in immune cell development and maturation, homeostasis, and disease pathogenesis [1–3]. In the central nervous system (CNS), cytokines have been shown to be released by microglia [4, 5], astrocytes [6–8], neurons [9–11], and endothelial cells [12], in response to microorganismal infections, injury, inflammation and in a number of neurological diseases [13–18].

In addition to their well-characterized immune-related functions, cytokines have also been reported to regulate synaptic transmission and neuronal excitability [19–23]. For example, TNF-α has been shown to be important for maintaining the strength of excitatory synapses and for reducing the strength of inhibitory synapses [24–27]; Interferon-γ augments GABAergic transmission in layer V pyramidal neurons [28, 29] and increases excitability of CA3 pyramidal neurons [30]; IL-1β inhibits long-term potentiation (LTP), reduces synaptic strength in hippocampal neurons [31–33] and increases the frequency of spontaneous excitatory postsynaptic currents (sEPSCs) in corticostriatal neurons [34]. In previous work, we showed that CCL2 (also known as Monocyte chemoattractant protein-1, MCP-1) elevates excitatory synaptic transmission in hippocampal CA1 and CA3 pyramidal neurons, L2/3 pyramidal neurons of the primary somatosensory cortex, as well as dentate gyrus granule cells [35]. This result is consistent with other reported functions of CCL2 in enhancing neuronal excitability in CA1 neurons [36], in increasing spontaneous EPSCs and potentiating AMPA- and NMDA-induced currents in lamina II neurons of the spinal cord [37], and in elevating Nav1.8 channel activity in primary sensory neurons [38].

The ability of CCL2 to promote excitatory synaptic transmission and neuronal excitability in multiple neuronal types suggested that a general mechanism may mediate this process. The strength of synaptic transmission is mostly dependent on the surface level of α-amino-3-hydroxy-5-methyl-4-isoxazolepropionic acid (AMPA) receptors. Phosphorylation of the C-terminal serine residues S845 and/or S831 of the AMPA receptor subunit GluA1 has been shown to potentiate synaptic transmission and promote surface delivery of AMPA receptors [39–47]. The S831 and S845 sites have been shown to be respectively regulated by protein kinases Calcium/calmodulin-dependent protein kinase II (CaMKII) and Protein kinase A (PKA), via changes in the intracellular concentrations of calcium and cyclic AMP (cAMP) [44–46, 48–52]. CCL2 signals mainly through binding to its cognate receptor C-C motif chemokine receptor 2 (CCR2), a G protein-coupled receptor (GPCR) reported to be widely expressed in the brain, in regions including the hippocampus, cerebral cortex, and hypothalamus [2, 53, 54]. GPCR activation, depending on whether it couples to Gα_q_, Gα_s,_ or Gα_i_, can modulate intracellular levels of calcium and/or cAMP. Could CCL2 binding to CCR2 affect AMPA receptor surface expression, and by extension, excitatory synaptic transmission, through changes in GluA1 phosphorylation and subsequent downstream signaling?

Here, we examined the contributions of CCL2 and CCR2 in regulating the surface expression of GluA1 using both *in vitro* and *in vivo* assays. We found that exogenous application of CCL2 in hippocampal culture neurons facilitated GluA1 surface expression, in a process dependent on CCR2, Gα_q_-signaling, and CaMKII activation, and with contributions from PKA signaling. We further showed that CCL2 promoted phosphorylation of GluA1 at S831 and S845 sites, in a CCR2-dependent fashion. Together, these results outline a mechanism through which CCL2 promotes the surface expression of GluA1 rapidly and effectively.

## Materials and Methods

### Animals

All animal procedures complied with the animal care standards set forth by the US National Institutes of Health and were approved by the Institutional Animal Care and Use Committee of the Institute of Neuroscience, Center for Excellence in Brain Science and Intelligence Technology, Chinese Academy of Sciences, and Peking University. Mice were kept on a C57BL/6 background and housed in cages containing corn bedding under a 12 h light-ON, 12 h light-OFF cycle, with food and water provided at libitum from the cage lid.

*Ccr2* knockout mice (*Ccr2* KO; *B6.129S4-Ccr2*^*tm1lfc/*^*J*; JAX strain 004999 | *Ccr2* KO) [55] on C57BL/6 background were obtained from the JAX laboratory. The P14 group consisted of P14-P15 mice, both male and female mice were used. The number of mice used in each experiment is indicated in figure legends.

Primary dissociated hippocampal cultures were prepared from P0 pups of Sprague-Dawley rats or mice on C57BL/6 background.

### Drugs Treatment

For treatment with CCL2, recombinant rat CCL2 (CCL2 (R&D, 3144-JE-050/CF) mouse CCL2 (R&D, 479-JE-050/CF) or human CCL2 (R&D, 279-MC-050/CF) was added, according to the origin of the cell type used; control was an equal amount of BSA (vehicle, R&D, RB02, 0.1% Bovine Serum Albumin in PBS, the dissolvent of CCL2). For lipopolysaccharides (LPS, Escherichia coli, serotype O111:B4, Sigma, Cat# L2630-25MG; 10 mg/kg) experiments, mice were intraperitoneally (i.p.) injected with a single dose of LPS, while littermate control animals received the same volume of saline (SA); mice were sacrificed 2 h post-injection.

### DNA Constructs

All DNA constructs encode vertebrate proteins expressed under the CAG promoter. pPiggyBac-hCCR2-P2A-mCherry was generated by subcloning hCCR2 from CCR2-Tango (a gift from Prof. Bryan L Roth, University of North Carolina, USA; Addgene: CCR2-Tango, RRID: Addgene_66239) into pPiggyBac-MRGPRX4-P2A-mCherry [56], replacing MRGPRX4 with hCCR2. SEP-GluA1, GluA2, and DsRed2 (gifts from Prof. Yong Zhang, Peking University) [57], and Nano-Luciferase [56] were as previously described. SEP-GluA1 consists of a pH-sensitive form of GFP (Super Ecliptic pHluorin, SEP) fused to the N-terminal regions of GluA1; SEP fluorescence is quenched in vesicles (low pH) and thus its fluorescence reflects the level of surface GluA1 expression.

### Real-Time Quantitative PCR (RT-qPCR)

RT-qPCR was carried out as described previously [35]. Briefly, total RNA was extracted from tissue (whole hippocampus) using TRIzol reagent (Invitrogen, 15596018). First-strand cDNA was generated using the M-MLV reverse transcriptase (Promega, M1701) according to the manufacturer’s protocols. RT-qPCR was performed using SYBR Green Master Mix (TaKaRa, RR420A) on LightCycler 480 (Roche Applied Science). All reactions were carried out in duplicates, and the comparative CT method was used. Primers used for RT-qPCR were as follows: *Ccl2*-F: CCGGCTGGAGCATCCACGTGT, *Ccl2*-R: TGGGGTCAGCACAGACCTCTCTCT; *Tnfα*-F: GACCCTCACACTCAGATCATCTTCT, *Tnfα*-R: CCTCCACTTGGTGGTTTGCT; *Il-1β*-F: CTCCATGAGCTTTGTACAAGG, *Il-1β*-R: TGCTGATGTACCAGTTGGGG; *Il-6*-F: ACACATGTTCTCTGGGAAATC, *Il-6*-R: AGTGCATCATCGTTGTTCATA; *Gria1*-F: CGAGTTCTGCTACAAATCCCG, *Gria1*-R: TGTCCGTATGGCTTCATTGATG; *Gria2*-F: AAAGAATACCCTGGAGCACAC, *Gria2*-R: CCAAACAATCTCCTGCATTTCC; *Gapdh*-F: CTGCCCAGAACATCATCCCT, *Gapdh*-R: TGAAGTCGCAGGAGACAACC.

### Fluo-8 Gα_q_-fluorescence Assay

A stable HEK293T cell line expressing pPiggyBac-hCCR2-P2A-mCherry was generated by transfecting the construct together with hyperactive PiggyBac transposase [58] and adding puromycin (1 mg/ml), using standard protocol [56]. The stable pPiggyBac-hCCR2-P2A-mCherry HEK293T was then reseeded in 96-well plates at a density of ~ 50,000 cells per well. The next day, cells were loaded with the Fluo-8, AM (4 μM, AAT Bioquest, 21083) for 1 h. The effects of recombinant human CCL2 and BSA (vehicle) were measured using the FLIPR TETRA system (PerkinElmer). Two independent cultures, each with 3 samples per condition were assayed.

### Gα_s_-luciferase Assay

HEK293T cells were seeded in 6-well plates; at a confluency of 80%, Nano-Luciferase [56] with/without pPiggyBac-hCCR2-P2A-mCherry were transfected using PEI MAX (Polysciences, 24765-100). One day later, cells were digested and reseeded in 96-well plates at a density of ~ 50,000 cells per well. Fresh culture medium containing forskolin (10 µmol/L), recombinant human CCL2, or BSA (vehicle) was added. Plates were incubated at 37 °C in 5% CO_2_ for 24 h, then 10 µL of culture medium from each well was mixed with 40 µL fresh culture medium and 50 µL assay buffer (containing coelenterazine, 20 µmol/L); after a further 5 min of incubation, luminescence was measured using EnVision plate reader (PerkinElmer). Three independent cultures, each with 3 samples per condition were assayed.

### Hippocampal Neuronal Culture and Transfection

Hippocampal neuron-glia co-cultures were prepared as previously described [59–62]. Briefly, primary hippocampal neuronal cultures were prepared from postnatal day 0 (P0) pups of Sprague-Dawley rats or mice on C57BL/6 background (males and females, randomly selected); hippocampi were dissected out, and dentate gyri were removed. ~ 120,000 cells were plated on Poly-D-lysine hydrobromide (PDL, Sigma, P7280) -coated 12 mm glass coverslips (Assistant, 01105209, Sondheim, Germany) in 24-well plates, or 35 mm glass bottom μ-Dishes (Ibidi, 81158, Martinsried, Germany). Culture medium contained Neurobasal medium (GIBCO, 10888022), B-27 (Invitrogen, 17504-044), 2 mmol/L Glutamax-I (Invitrogen, 35050061), and 2.5% FBS (HyClone, Logan, UT, USA). Cells were cultured at 37 °C in 5% CO_2_. On the third *day in vitro* (DIV 3), when the astrocytes had grown sufficiently to form a monolayer covering the entire coverslip, cells were treated with the mitotic inhibitor FUDR (5-fluoro-2′-deoxyuridine, Sigma, F0503). Calcium phosphate transfections were carried out at DIV 7 using standard protocols [63].

As previously characterized [62, 64, 65], pyramidal neurons account for ~ 90% of total cultured neurons, with the rest being GABAergic interneurons. Pyramidal neurons and GABAergic neurons have distinctive morphologies, with the somata of the latter being more fusiform or polygonal in shape and having fewer primary dendrites [65]. Pyramidal neurons were selected for further analyses based on these criteria.

### Immunocytochemistry, Pharmacology, and Data Analysis

The following antibodies were used: Anti-GluA1 (N-terminus, clone RH95, Millipore, MAB2263, RRID: AB_11212678; 1:200), MAP2 (Millipore, AB5622, RRID: AB_91939; 1:1000), Donkey anti-Mouse Alexa Fluor 488 (Thermo Fisher Scientific, R37114, RRID: AB_2556542; 1:1000), Goat anti-Rabbit Alexa Fluor 568 (Thermo Fisher Scientific, A78955, RRID: AB_2925778; 1:1000). DIV 12 culture hippocampal neurons were treated with conditional medium containing anti-N-GluA1 antibody and CCL2 (100 ng/mL), or equal amount of BSA (vehicle), and incubated for 20 min, at 37 °C and in 5% CO_2_. For pretreatment experiments, RS504393 (R&D, 2517, 10 µmol/L), U73122 (Sigma, U6756, 5 µmol/L), KN-93 (Tocris, 1278, 20 µmol/L), PKI 14-22 (Tocris, 2546, 5 µmol/L) or DMSO (vehicle, Sigma, D2650; 0.1 % v/v) were added 30 min before BSA/CCL2 application. After drug treatment, neurons were washed twice with warm PBS, and fixed using cold 4% PFA for 15 min at room temperature, permeabilized, and processed for immunocytochemistry according to standard protocols. Images were acquired on a Nikon A1 confocal microscope with a Plan Apo 60× oil-immersion objective (N.A. = 1.40) at 0.5 μm Z intervals. Maximum projection images were analyzed using Image-Pro Plus 6.0 (Media Cybernetics, Rockville, MD, USA). The total surface area or intensity of GluA1 of each image frame was normalized to that of MAP2.

All images were coded using random sequences (https://www.random.org/sequences/) at the time of acquisition and analyzed blindly to the experimental condition. For example, images, and brightness/contrast were adjusted within linear ranges using Fiji/ImageJ when necessary. Control and experimental conditions were adjusted using the same parameters.

### Live Imaging and Data Analysis

Live imaging experiments were carried out on a Nikon A1 confocal microscope with a Plan Apo 60× oil-immersion objective (N.A. = 1.40), at 2× optical zoom and 0.5 μm Z intervals; images were acquired at 5-min intervals. DIV 7 neurons were transfected with SEP-GluA1, GluA2, and DsRed2 (at a ratio of 9:9:2) to mimic endogenous GluA subunit ratios [57]. Live imaging was performed at DIV 14–15. Neurons grown on 35-mm glass bottom dishes were placed in a Stage Top Incubator (Tokai Hit, Japan), and the environment was maintained at 37 °C and 5% CO_2_. Recombinant rat CCL2 (100 ng/mL) or BSA (vehicle) was added to the culture medium after baseline images were acquired. Neurons were pretreated with RS504393 (R&D, 2517, 10 µmol/L) for 30 min before BSA/CCL2 application. Images were analyzed using Image-Pro Plus 6.0 (Media Cybernetics, Rockville, MD, USA).

Analysis of surface SEP-GluA1 expression was limited to spines, using the following criteria: (1) located on secondary dendrites; (2) stable baseline; (3) presented in at least 4 images (6 total); less than 5 µm in length. The region of interest (ROI) was marked in the morphology channel (DsRed2), and the area and total intensity of the SEP-GluA1 channel were ratioed to that of the DsRed2 channel. For quantification of before and after treatment, “before” included the − 5 min and 0 min time points, while “after” included the 5 min, 10 min, 15 min, and 20 min time points. 4–5 independent culture preparations were used per condition.

### Calcium Imaging in Cultured Hippocampal Neurons

Cultured hippocampal neurons were infected with AAV2/9-hSyn-jGCaMP7b-WPRE-pA (0.5 μL, 1.42 × 10^13^ TU/mL, S0591-9, Shanghai Taitool Bioscience) at DIV 4, and imaged at DIV 14. Imaging was carried out on a Nikon A1 confocal microscope, with a Plan Apo 20x objective (N.A. = 0.75); A perfect focus system (PFS) was used, and images were acquired at 2 (baseline), 10 and 15 min, continuously for 90 s at 1.33 Hz at each data point  (~ 120 frames). BSA (vehicle) or recombinant rat CCL2 (100 ng/mL) was perfused from 3 min to 15 min in the extracellular solution contained (in mmol/L; NaCl 129, KCl 5, glucose 30, HEPES 25, CaCl_2_ 2, and MgCl_2_ 1; pH 7.3; 310 mOsm).

Calcium transients were identified using PeakCaller [66]. Parameter settings were as follows: required rise = 80% absolute; max. lookback = 10 pts; required fall = 80% absolute; max. lookahead = 10 pts; trend control = no trend. Average peak amplitudes and frequencies of calcium transients were normalized to baseline fluorescence. All image analyses were carried out with no post-acquisition modifications. Example images under control and experimental conditions were adjusted with the same parameters.

### Electrophysiology in Cultured Hippocampal Neurons

Electrophysiology was performed as previously described [67]. Briefly, whole-cell mEPSC recordings of hippocampal neuronal cultures (DIV 8-10) were made with a MultiClamp 700B amplifier (Molecular Devices, Sunnyvale, CA, USA). Neurons were held at − 70 mV in a voltage clamp. Signals were filtered at 2 kHz and sampled at 10 kHz using Digidata 1550B (Molecular Devices). TTX (0.5 μmol/L) and Gabazine (10 μmol/L) were added to artificial cerebrospinal fluid (aCSF) to block Na^+^ channels and GABA_A_ receptors, respectively. The aCSF contained (in mmol/L): NaCl 125, KCl 2.5, NaH_2_PO_4_ 1.3, MgCl_2_ 1.3, CaCl_2_ 2, NaHCO_3_ 25, and Glucose 20. One neuron from each coverslip was recorded, first perfused in BSA (vehicle) for 4–5 min and then in CCL2 (100 ng/mL).

Data were analyzed in MiniAnalysis (Synaptosoft, Fort Lee, NJ) with an amplitude detection threshold of 5 pA. Data were analyzed blinded to treatment. During recording, a brief hyper-polarization (− 10 mV, 100 ms) was given to monitor series and input resistances every 10 s. Neurons with series resistance of more than 20 MΩ or changes of series resistance of greater than 20% were excluded from analysis.

### Western Blots

Western blots were performed as previously described [35]. Mice were deeply anesthetized with 0.7% sodium pentobarbital at 0.14 g/kg body weight. The brains were removed quickly, and the hippocampi were dissected. Brain samples were homogenized with a motorized tissue grinder in HEPES buffer (0.32 mol/L sucrose and 4 mmol/L HEPES, pH 7.4) containing freshly added protease inhibitor cocktail tablets (Roche, 04693132001) and phosphatase inhibitor cocktail tablets (Roche, 4906845001). The total homogenates were centrifuged at 1000 *g* for 10 min to remove the nuclear fraction. The supernatant was collected and then centrifuged at 10,000 *g* for 20 min to yield the crude membrane fraction (P2).

For purifying synaptosome membrane fractions, the P2 fraction was resuspended with HEPES buffer (0.32 mol/L sucrose and 4 mmol/L HEPES, pH 7.4) and centrifuged at 10,000 *g* for 15 min to yield the washed P2, followed by hypotonic treatment (ddH_2_O treated with protease inhibitor cocktail tablet) to rupture vesicles. 1 mol/L HEPES (pH 7.4) was quickly added to maintain the osmotic pressure of the solution at 4 mmol/L HEPES; solutions were incubated on ice for 1 h for thorough lysis. The lysed solution was centrifuged at 25,000 *g* for 20 min to yield the pellet (P3, lysed synaptosomal membrane fraction). The P3 then was resuspended and added onto a discontinuous sucrose gradient (top to bottom, 0.85/1.0/1.2 mol/L sucrose in 4 mmol/L HEPES with protein inhibitor), followed by ultracentrifugation at 30,000 r/min for 2 h at 4 °C; the fraction between 1.0 mol/L and 1.2 mol/L was collected as P4. 15 µg of samples (P2 or P4) were loaded per lane; PVDF membranes were blocked in 5% BSA blocking solution, and incubated with primary antibodies overnight at 4 °C; HRP-conjugated secondary antibodies incubation was 1 h at room temperature. For quantification of phosphorylation levels, the phosphorylation-site-specific antibodies were stripped from the membranes using stripping buffer (Thermo Scientific, 46430) for 30 min at room temperature, followed by blocking in 5% BSA blocking solution for 1 h, and then reprobed with anti-total protein antibodies.

The following primary antibodies were used: GluA1 (Millipore, AB1504, RRID:AB_2113602; 1:1000), GluA1 S831 (Abcam, ab109464, RRID:AB_10862154; 1:1000), GluA1 S845 (Abcam, ab76321, RRID:AB_1523688; 1:1000), GluA2 (Millipore, MAB397, RRID:AB_2113875; 1:1000), GluA2 S880 (Abcam, ab52180, RRID:AB_880227; 1:1000), CaMKII (Invitrogen, MA1-048, RRID:AB_325403; 1:1000), CaMKII T286 (Abcam, ab5683, RRID:AB_305050; 1:1000), PKA (Cell signaling, #4782, RRID:AB_2170170; 1:1000), PKA T197 (Cell signaling, #4781, RRID:AB_2300165; 1:1000), and GAPDH (Kangchen Biotech, KC-5G4, RRID:AB_2493106; 1:5000). The following secondary antibodies were used: HRP conjugated goat anti-mouse IgG Antibody (ZSGB-Bio, ZB-2305; RRID: AB_2747415; 1:2500) and HRP conjugated goat anti-mouse IgG Antibody (ZSGB-Bio, ZB-2301, RRID: AB_2747412; 1:2500). Signals were visualized using an ECL Plus kit (PE0010, Solarbio, China). Blots were quantitated using Fiji/ImageJ (N.I.H, Bethesda, MD) and normalized to the gray value of GAPDH or total protein (for phosphorylation-site-specific antibodies).

### Tail Suspension Test (TST)

A tail suspension test was carried out as previously described [35]. P14 mice were suspended 30 cm above the floor, by tape placed about 1 cm from the end of the tail. Videos were recorded from the mouse’s ventral side. Immobility percentage within 6 min was analyzed blinded to the experimental condition using the “FST/TST” module in the SMART video tracking system (Panlab 3.0, Harvard Apparatus, Holliston, MA, USA).

### Statistical Analysis

Statistical analyses were performed using GraphPad Prism 9 (GraphPad Software, La Jolla, CA, USA). Data were analyzed blinded to the experimental condition. Gaussian distribution of the data was assessed using the Shapiro-Wilk normality test, KS normality test, Anderson-Darling test, or D’Agostino & Pearson normality test. If data passed the Gaussian distribution test, parametric tests (paired two-tailed *t*-test or unpaired two-tailed *t*-test for two groups; or one-way ANOVA with Tukey’s post hoc test for three or more groups) were used; otherwise, nonparametric tests (Mann-Whitney for unpaired two groups, Wilcoxon matched-pairs signed rank test for paired two groups) were used. For surface GluA1 staining, SEP-GluA1 live imaging, and calcium imaging, two-way ANOVA followed by the Bonferroni *post hoc* test was used. Cumulative distributions were tested using the Kolmogorov-Smirnov test. For western blotting experiments and tail suspension test, n represents the number of mice; for reporter assays, *n* represents independent wells; for immunocytochemistry and electrophysiology, and calcium imaging experiments, *n* represents the number of neurons; for SEP-GluA1 imaging, *n* represents the number of spines. At least three mice or independent cultured neuronal preparations were used per experimental condition. Results are shown as mean ± SEM and statistical significance was set at *P < 0.05, **P < 0.01, ***P < 0.001; n.s., not significant.

## Results

### Exogenous CCL2 Application Elevates SEP-GluA1 Level

**Fig. 1:**
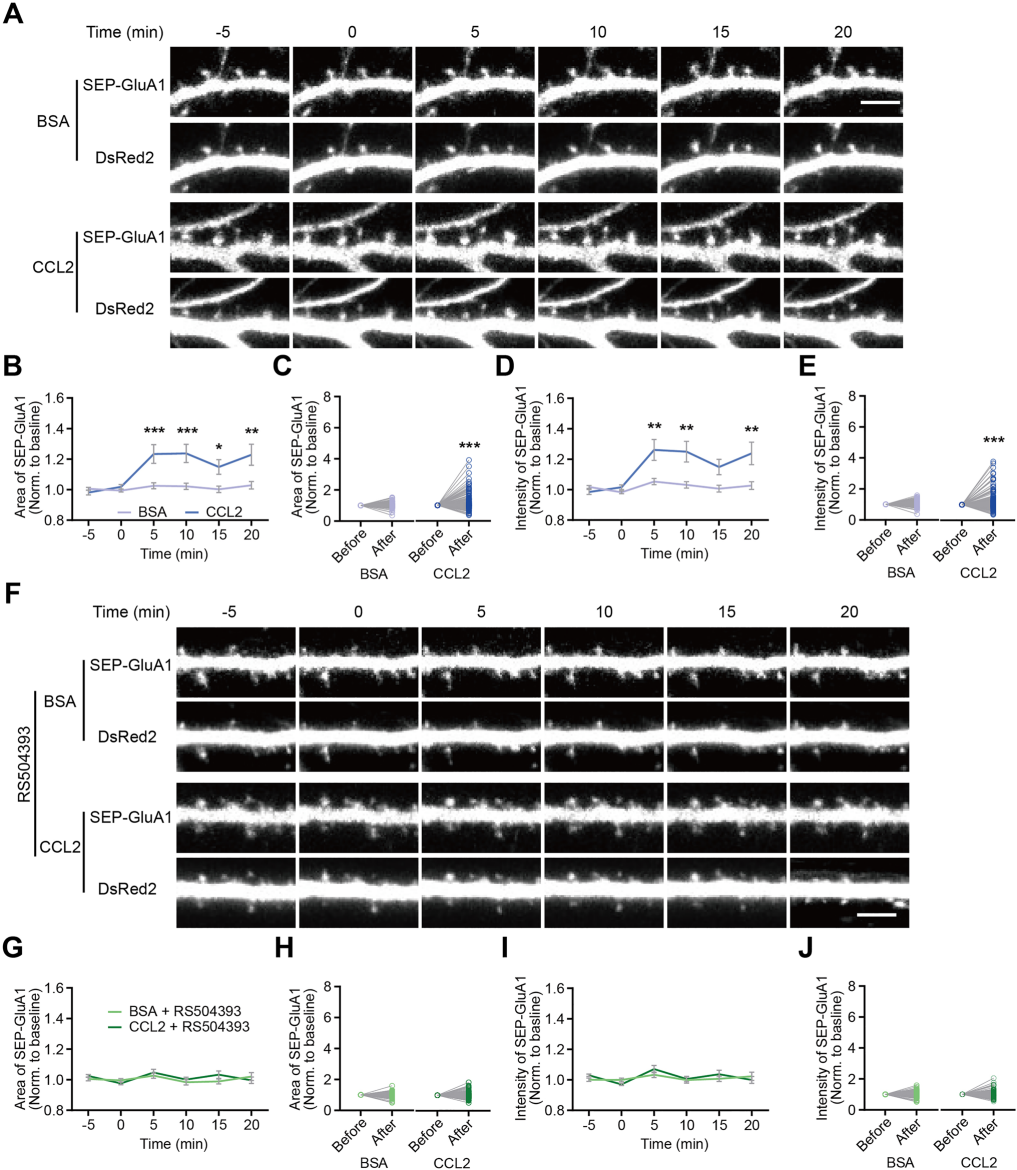
CCL2-induced elevation in surface SEP-GluA1 expression in rat hippocampal neurons requires CCR2 signaling. **A**, **F** Representative time-lapse images showing expression of SEP-GluA1 and DsRed2 in spines upon BSA, CCL2, and/or RS504393/DMSO (vehicle) application, conditions as indicated. **B**, **D** Changes in SEP-GluA1 area (**B**) and intensity (**D**) upon CCL2 application (BSA, *n =* 129 spines from 6 neurons; CCL2, *n =* 142 spines from 9 neurons; significance as indicated on the graph). **C**, **E** The effects of CCL2 on spine SEP-GluA1 area (**C**, BSA, *P* = 0.42; CCL2, *P* < 0.001) and intensity (**E**, BSA, *P* = 0.10; CCL2, *P* < 0.001), respectively. **G**, **I** Pretreatment of RS504393/DMSO (vehicle) blocked the effects of CCL2 application on SEP-GluA1 area (**G**) and intensity (**I**) (BSA, *n =* 123 spines from 6 neurons; CCL2, *n =* 134 spines from 6 neurons; significance as indicated on the graph). **H**, **J** The effects of CCL2 on SEP-GluA1 area (**H**, BSA, *P* = 0.85; CCL2, *P* = 0.86) and intensity (**J**, BSA, *P* = 0.34; CCL2, *P* = 0.55), in neurons pretreated with RS504393/DMSO. Wilcoxon matched-pairs signed rank *t*-test was used for (**C**, **E**, **H**, **J**); two-way ANOVA followed by Bonferroni’s *post hoc* test for (**B**, **D**, **G**, **I**). Each data point represents one spine. Scale bar, 5 μm. In this and all subsequent figures, data are presented as mean ± SEM. n.s., not significant; *P < 0.05, **P < 0.01, ***P < 0.001.

The chemokine CCL2 has been previously reported to modulate neuronal excitability and synaptic transmission, likely through post-synaptic mechanisms [35–38, 68–70]. However, the molecular mechanism through which it is achieved remains unclear. Here, we used primary dissociated hippocampal neuronal cultures to examine the underlying mechanism, focusing on the regulation of surface AMPA receptor level. To start, we confirmed that perfusion of CCL2 (100 ng/mL; Ctrl is BSA) significantly elevated the frequency of mEPSCs (Fig. S1), consistent with its effects on CA1 pyramidal neurons in acute brain slices [35]. We next transfected cultured hippocampal neurons with SEP-GluA1, consisting of a pH-sensitive form of GFP (Super Ecliptic pHluorin, SEP) fused to the N-terminal regions of GluA1, which allows direct visualization of surface GluA1 in real-time [57, 71]; DsRed2 was used as a morphology marker (Fig. 1A). CCL2 application effectively increased the area (Fig. 1B) and intensity (Fig. 1D) of SEP-GluA1 puncta, with significant changes observed 5 min following CCL2 application. The averaged fluorescence changes before and after CCL2 application were also significantly different, both in terms of area (BSA, *P* = 0.42; CCL2, *P* < 0.001; Fig. 1C) and intensity (BSA, *P* = 0.10; CCL2, *P* < 0.001; Fig. 1E). Importantly, CCL2-induced increase in SEP-GluA1 expression was effectively blocked by the competitive CCR2 antagonist RS504393 (Fig. 1F), both when measuring SEP-GluA1 puncta area (BSA, *P* = 0.85; CCL2, *P* = 0.86; Fig. 1G, H) and intensity (BSA, *P* = 0.34; CCL2, *P* = 0.55; Fig. 1I, J). Together, the above results demonstrated that CCL2 rapidly induced surface expression of GluA1 in a CCR2-dependent manner.

### CCL2 Application Promotes GluA1 Membrane Trafficking via CCR2 Signaling in Cultured Hippocampal Neurons

**Fig. 2:**
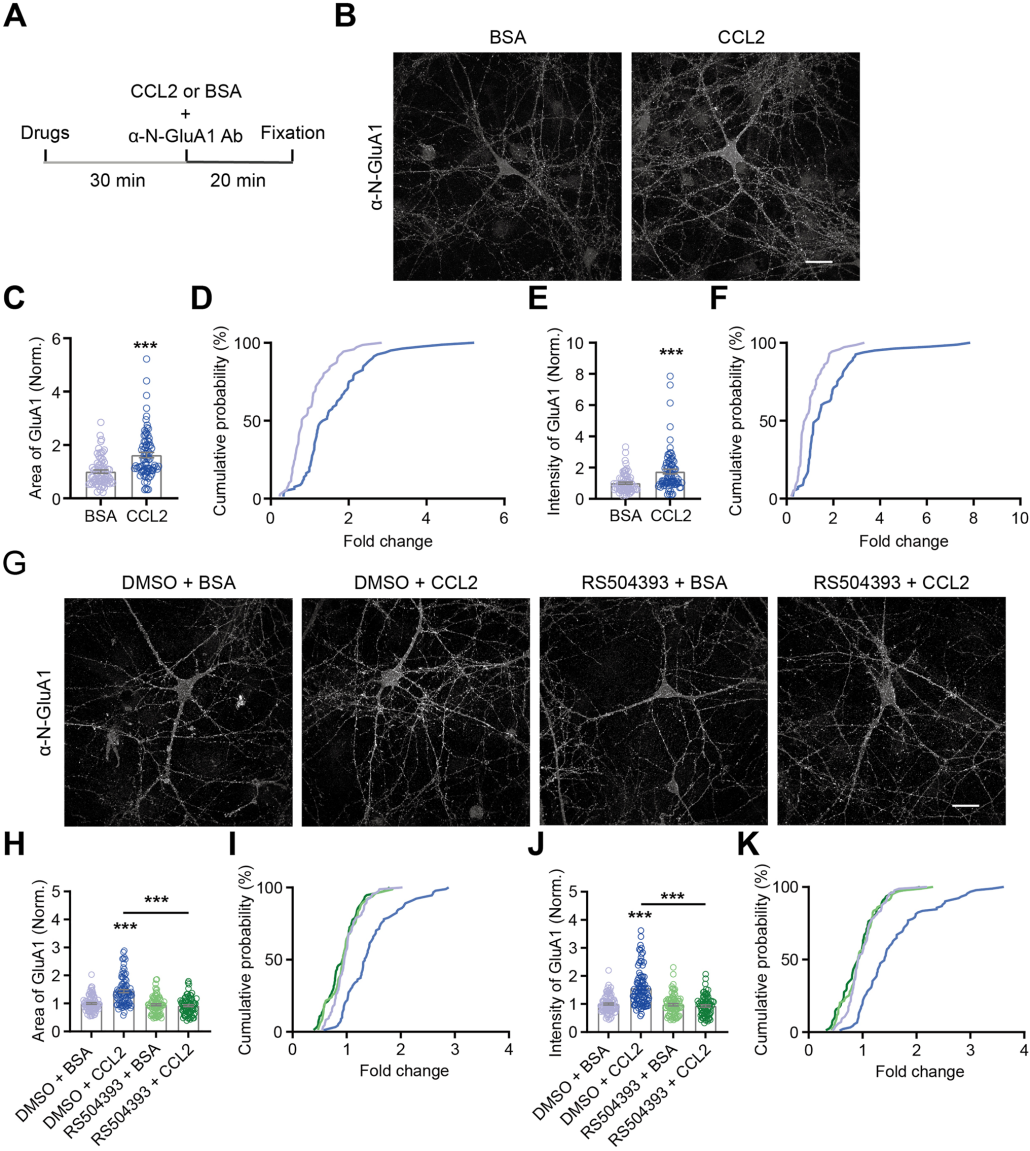
CCL2 regulates surface GluA1 levels in cultured rat hippocampal neurons via CCR2 signaling. **A** Schematic showing the flow of surface GluA1 staining. **B** Representative images of surface GluA1 immunostaining following BSA (left, control) or CCL2 (right) treatment. **C**, **E** Quantification of surface GluA1 area (**C**) and intensity (**E**) (BSA, *n =* 71; CCL2, *n =* 80; significance as indicated on the plot). **D**, **F** Cumulative probability distribution of area (**D**) and intensity (**F**) (BSA *vs.* CCL2, *P* < 0.001 for both). **G** Representative images of surface GluA1 immunostaining following CCL2/BSA and/or RS504393/DMSO (vehicle) treatment, conditions as indicated. **H**, **J** Quantification of the area (**H**) and intensity (**J**) of surface GluA1, conditions as indicated. CCL2 increased surface GluA1 level, an effect blocked by the CCR2-antagonist RS504393 (RS) (DMSO + BSA, *n =* 85; DMSO + CCL2, *n =* 91; RS + BSA, *n =* 79; RS + CCL2, *n =* 76; significance as indicated on plot). **I**, **K** Cumulative probability distribution of surface GluA1 area (**I**) and intensity (**K**) (for both plots, DMSO + CCL2 *vs.* all others: *P* < 0.001, no significant differences among other 3 conditions). Each data point represents one neuron. Mann-Whitney test was used for (**C**, **E**); Kolmogorov-Smirnov test for (**D**, **F**, **I**, **K**); two-way ANOVA followed by Bonferroni’s *post hoc* test for (**H**, **J**). Scale bar, 25 μm.

We further confirmed the above results using surface GluA1 staining. DIV 12 hippocampal neuronal cultures were treated with CCL2 (100 ng/mL; Ctrl is BSA) and antibody against N-terminal region of GluA1 for 20 min at 37 °C to label surface GluA1; cells were then fixed and processed for immunocytochemistry (Fig. 2A). CCL2 application significantly increased both the total area (BSA, 1.00 ± 0.06; CCL2, 1.62 ± 0.10; *P* < 0.001; Fig. 2B–D) and total intensity (BSA, 1.00 ± 0.07; CCL2, 1.75 ± 0.15; *P* < 0.001; Fig. 2E, F) of surface GluA1. Pretreating neurons for 30 min with the competitive CCR2 antagonist RS504393, prior to CCL2 application, abolished the upregulation of surface GluA1 area (DMSO + CCL2 *vs.* all others, *P* < 0.001; no significant differences among other 3 conditions; Fig. 2G–I) and intensity (Fig. 2J, K) induced by CCL2.

**Fig. 3:**
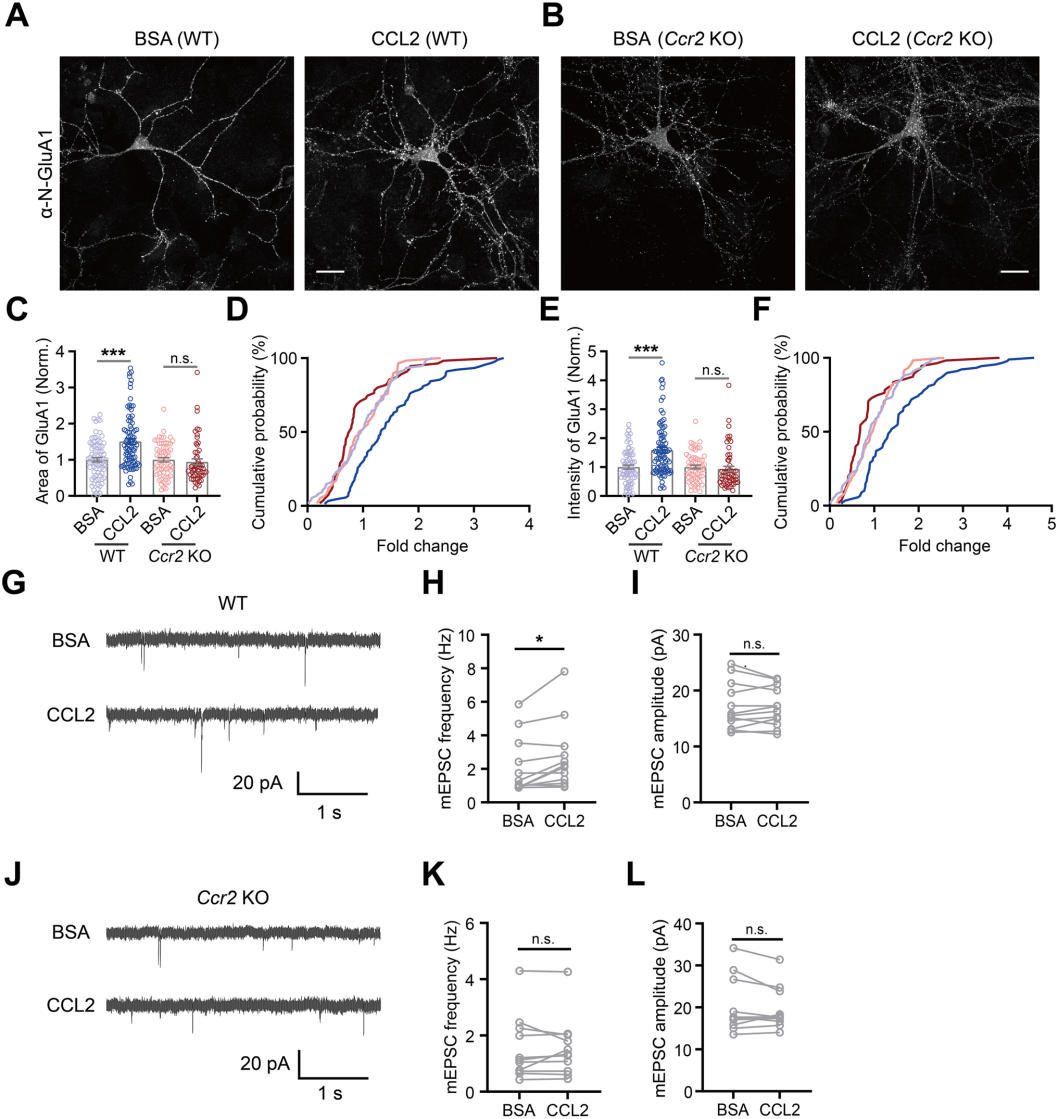
CCR2 is required for CCL2-dependent upregulation of excitatory synaptic transmission and GluA1 trafficking in cultured mouse hippocampal neurons. **A**, **B** Representative images of surface GluA1 immunostaining following BSA/CCL2 treatment of cultured hippocampal neurons from WT **A** or *Ccr2* knockout mice (**B**). **C**, **E** Quantification of surface GluA1 area (**C**) and intensity (**E**) (WT: BSA, *n =* 75; CCL2, *n =* 89; *Ccr2*^*-/-*^: BSA, *n =* 58; CCL2, *n =* 55; significance as indicated on the plot). **D**, **F** Cumulative probability distribution of area (**D**) and intensity (**F**) (WT: Area, BSA *vs.* CCL2, *P* < 0.001; Intensity, BSA *vs.* CCL2, *P* < 0.001; *Ccr2*^*-/-*^: Area, BSA *vs.* CCL2, *P* = 0.11; Intensity, BSA *vs.* CCL2, *P* < 0.05). **G**–**I** Representative traces (**G**) and summary data (**H**, **I**) of the effects of CCL2 on mEPSC frequency and amplitude of cultured hippocampal neurons from WT mice (Frequency: BSA, 2.10 ± 0.49 Hz; CCL2, 2.68 ± 0.58 Hz, *P* < 0.05; Amplitude: BSA, 17.20 ± 1.21 pA; CCL2, 17.10 ± 1.012 pA, *P* = 0.81; *n =* 12 for both conditions). **J**–**L** Representative traces (**J**) and summary data (**K**, **L**) of the effects of CCL2 on mEPSC frequency and amplitude of cultured hippocampal neurons from *Ccr2* knockout mice (Frequency: BSA, 1.54 ± 0.34 Hz; CCL2, 1.56 ± 0.32 Hz, *P* = 0.89; Amplitude: BSA, 20.26 ± 1.99 pA; CCL2, 19.59 ± 1.52 pA, *P* = 0.46; *n =* 11 for both conditions). Each data point represents one neuron. Mann-Whitney test was used for (**C**, **E**); Kolmogorov-Smirnov test for (**D**, **F**); Wilcoxon matched-pairs signed rank *t*-test was used for (**H**, **L**); paired two-tailed *t-*test for (**I**, **K**). Scale bar, 25 μm.

To further confirm that CCR2 mediated this effect, we prepared cultured hippocampal neurons from *Ccr2* knockout mice (*Ccr2*^*−/−*^) (Fig. 3). The effects of CCL2 application on increasing surface GluA1 area (BSA, 1.00 ± 0.06; CCL2, 1.51 ± 0.08; *P* < 0.001; Fig. 3C, D) and intensity (BSA, 1.00 ± 0.07; CCL2, 1.59 ± 0.10; *P* < 0.001; Fig. 3E, F) in wildtype mouse neuronal cultures, were completely blocked in cultures prepared from *Ccr2*^*−/−*^ mice [area (BSA, 1.00 ± 0.06; CCL2, 0.94 ± 0.08; *P* = 0.19; Fig. 3C, D); intensity (BSA, 1.00 ± 0.07; CCL2, 0.93 ± 0.09; *P* = 0.10; Fig. 3E, F)]. Consistently, the effects of CCL2 in significantly elevating mEPSC frequency (Fig. 3G–I) of wildtype cultured hippocampal neurons were also blocked in cultured neurons from *Ccr2*^*−/−*^ mice (Fig. 3J–L). Together, these results demonstrated that CCL2 promotes surface GluA1 expression via CCR2 signaling.

### GPCR-signaling Downstream of CCR2

**Fig. 4:**
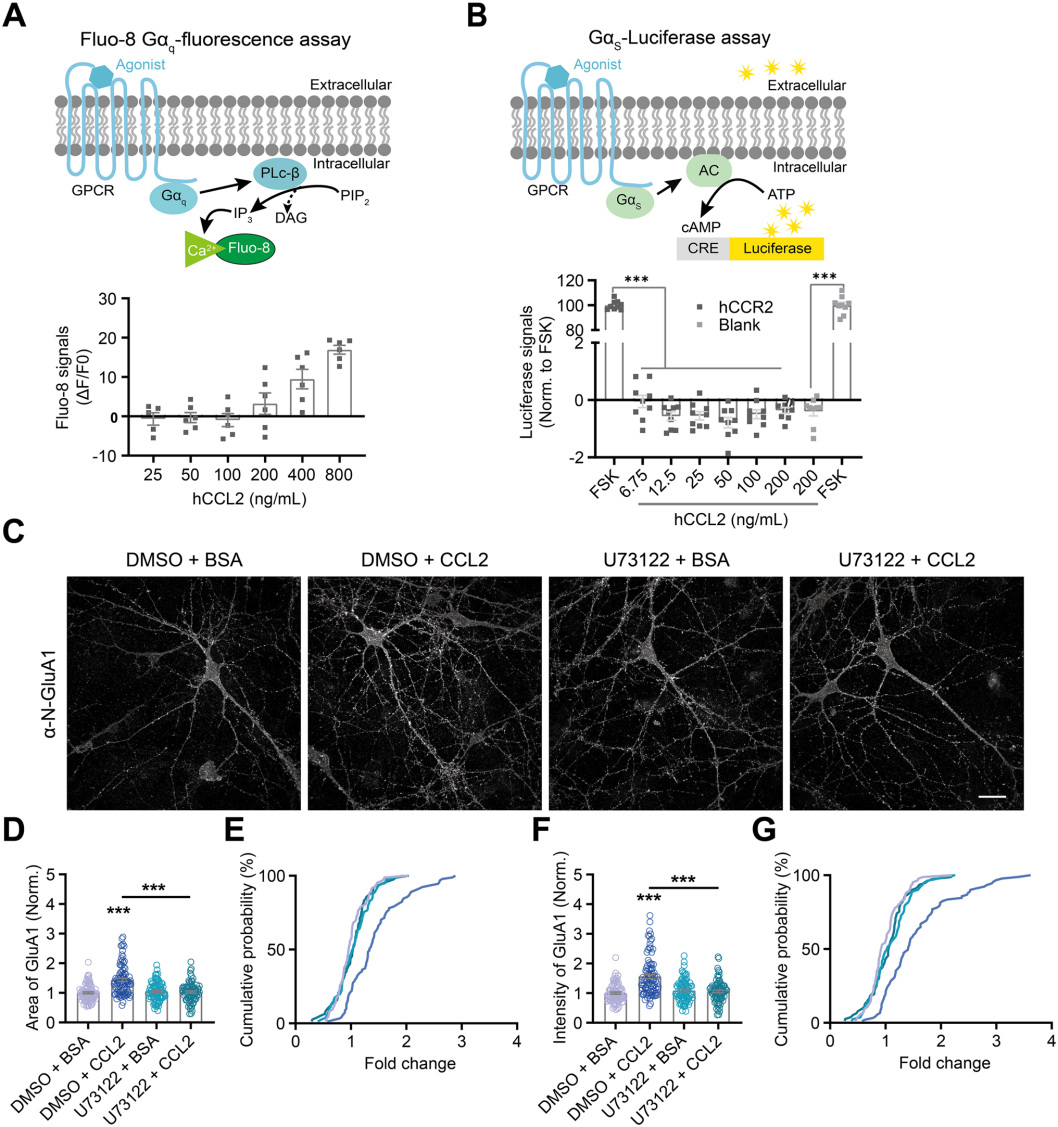
CCR2 signals through Gα_q_. **A** Top, schematic of calcium imaging using the Fluo-8 Gα_q_-fluorescence reporter assay; Bottom, hCCL2 increased intracellular calcium level via Gα_q_ in a dose-dependent way. **B** Top, schematic of the Gα_s_-Luciferase assay; bottom, hCCL2 did not significantly affect Gα_s_ activity, while positive control forskolin did. **C** Representative images of surface GluA1 immunostaining in rat hippocampal neurons following CCL2/BSA and/or U73122/DMSO (vehicle) treatment, conditions as indicated. **D**, **F** Quantification of surface GluA1 area (**D**) and intensity (**F**), conditions as indicated. CCL2 treatment increased area of surface GluA1, an effect blocked by U73122 (DMSO + BSA, *n =* 85; DMSO + CCL2, *n =* 91; U73122 + BSA, *n =* 81; U73122 + CCL2, *n =* 77; significance as indicated). **E**, **G** Cumulative probability distribution of area (**E**) and intensity (**G**) (for both plots, DMSO + CCL2 *vs.* all others: *P* < 0.001, no significant differences among other 3 conditions), respectively. Data for DMSO + BSA and DMSO + CCL2 in 4D and 4F are the same as that in Figures 2H and J, respectively. Each data point represents one independent sample (**A**, **B**) or one neuron (**C**–**G**). one-way ANOVA followed by Tukey’s *post hoc* test for (**B**); Kolmogorov-Smirnov test for (**E**, **G**); two-way ANOVA followed by Bonferroni’s *post hoc* test for (**D**, **F**). Scale bar, 25 μm.

How does CCR2 signal downstream to regulate AMPA receptor level? CCR2 is a GPCR that can signal through various second messengers [72, 73]. Overlapping those known to affect AMPA receptor levels, we focused on the regulation of intracellular Ca^2+^ levels through Gα_q_ signaling and the regulation of cAMP levels through Gα_s_ [42, 74, 75]. For calcium imaging, we constructed a stable cell line expressing hCCR2 in HEK293T cells, which does not endogenously express this receptor [76]; hCCL2 elevated intracellular Ca^2+^ level in this cell line in a dose-dependent way (Fig. 4A). In the Gα_s_-Luciferase assay [77], hCCL2 had no significant effects on intracellular cAMP level, while the positive control forskolin did (Fig. 4B). Together, these results suggested that CCR2 couples to Gα_q_, but not Gα_s_, in HEK293T cells, consistent with previous reports [53, 78].

**Fig. 5:**
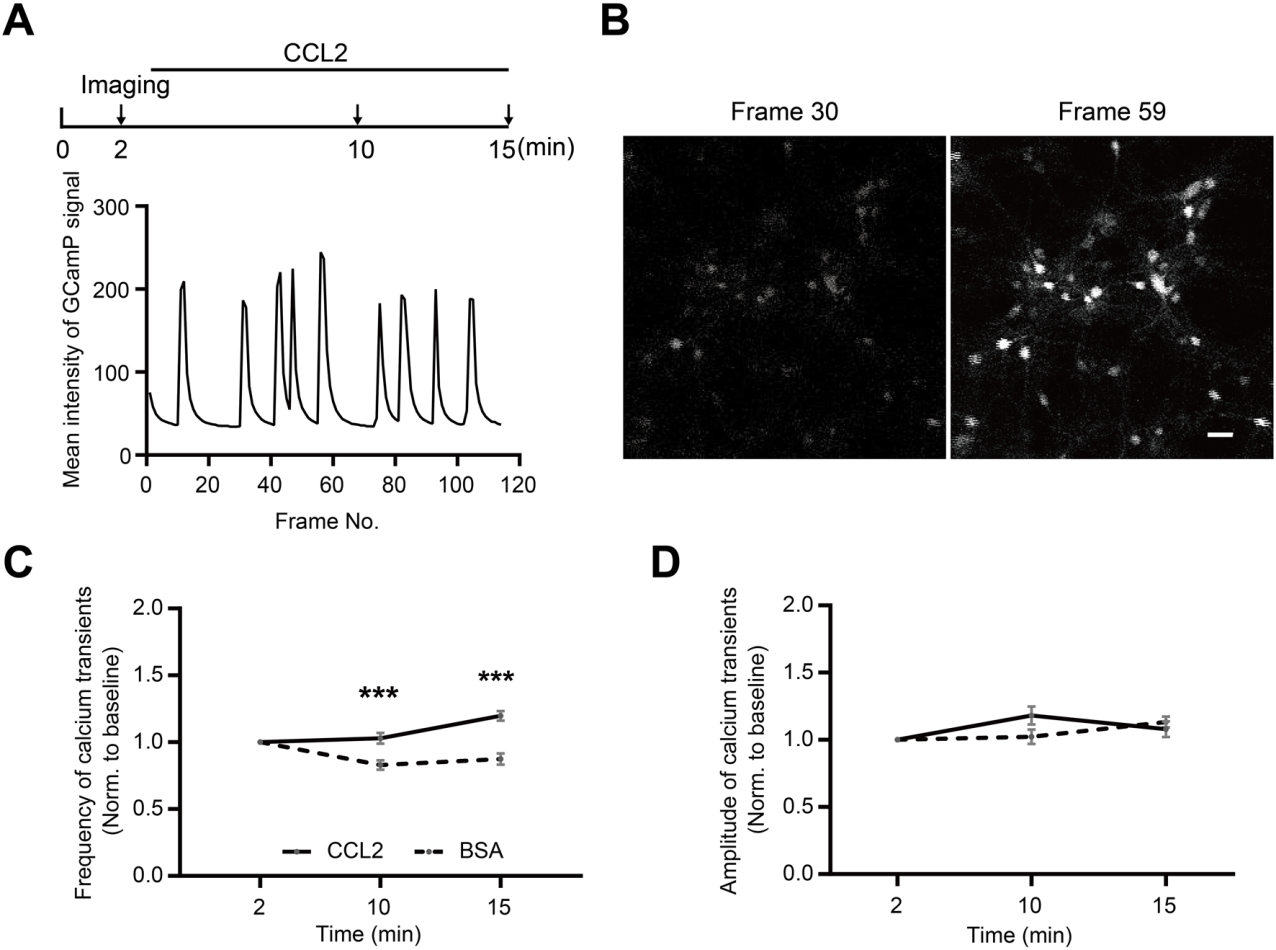
CCL2 increases the frequency of calcium transients in cultured rat hippocampal neurons. **A**, **B** Schematic of experimental procedure (A, upper), representative whole frame GCaMP signal quantification (A, lower) from a single time point, and example images showing baseline (frame 30) and peak (frame 59) calcium signals (B). **C**, **D** Summary data of the effect of CCL2 on the frequency (C) and amplitude (D) of calcium transients (BSA, *n =* 83; CCL2, *n =* 110; Frequency: 2 min, *P* > 0.99; 10 min, *P* < 0.001; 15 min, *P* < 0.001; Amplitude: 2 min, *P* > 0.99; 10 min, *P* = 0.06; 15 min, *P* > 0.99). “n” represents the number of neurons. Two-way ANOVA followed by Bonferroni’s *post hoc* test for (**C**, **D**). Scale bar, 50 μm.

What about neurons? Pre-incubation of cultured hippocampal neurons for 30 min with U73122, an inhibitor of phospholipase C (PLC), which signals downstream of Gα_q_, effectively abolished the effects of CCL2 in promoting GluA1 surface expression (Fig. 4C–G). Quantification showed a significant reduction in surface GluA1 area (DMSO + CCL2 *vs.* all others, *P* < 0.001; no significant differences among other 3 conditions; Fig. 4D, E) and intensity (Fig. 4F, G). Gα_q_ activation elevates intracellular calcium. Consistently, in cultured neurons expressing GCaMP7b, CCL2 application significantly increased the frequency of calcium transients, without affecting the average amplitude (Fig. 5A–D). Together, these results suggest that CCR2 signals via Gα_q_ and elevates intracellular calcium.

### Protein Kinases Mediate CCL2-induced GluA1 Membrane Trafficking

**Fig. 6:**
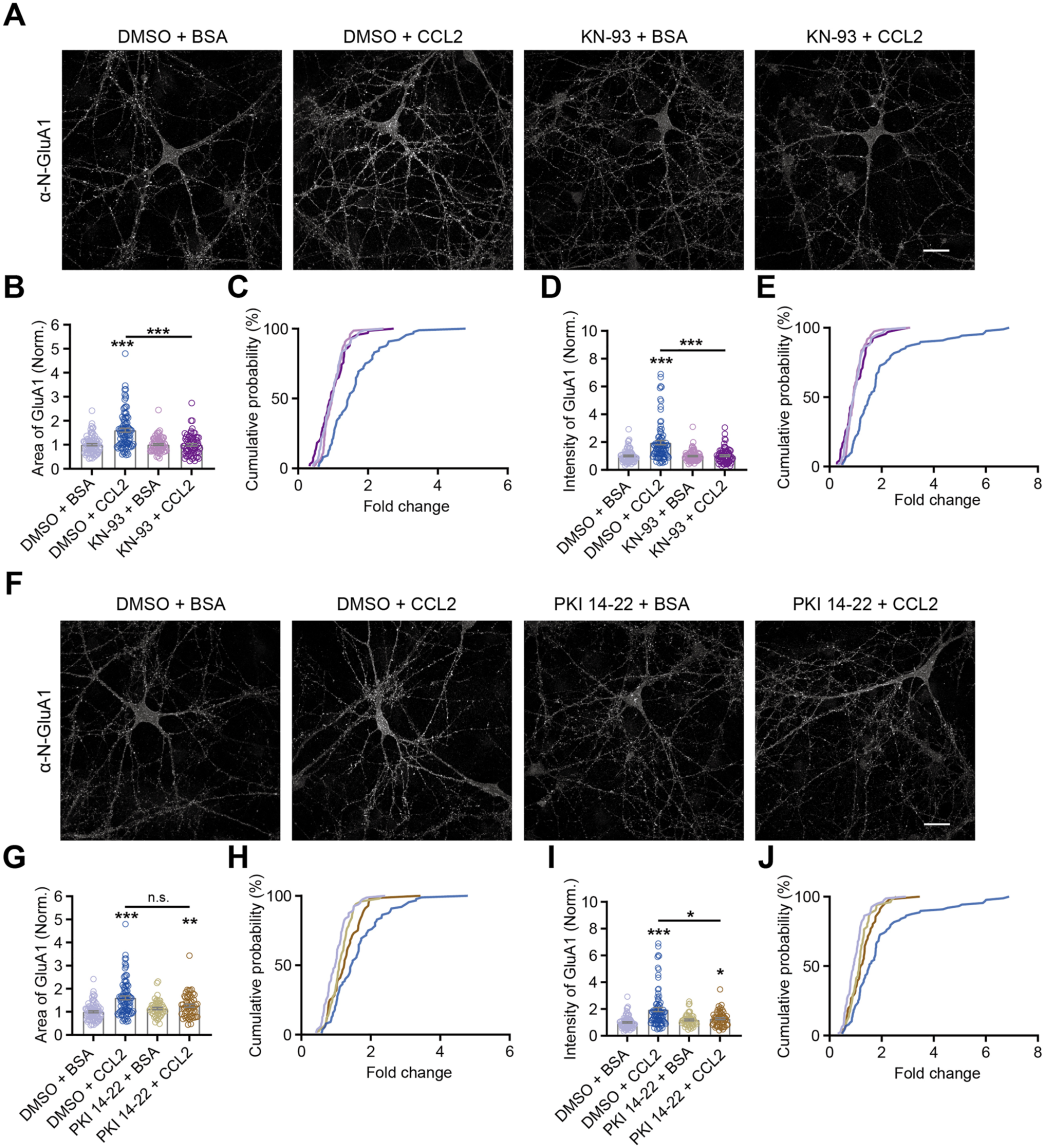
CCL2 elevates GluA1 surface expression via CaMKII signaling. **A**, **F** Representative images of surface GluA1 immunostaining upon application of CCL2/BSA, together with KN-93 (KN)/DMSO (vehicle) or PKI14-22 (PKI)/DMSO (vehicle), conditions as indicated. **B**, **D**, **G**, **I** Quantification of surface GluA1 area (**B**, **G**) and intensity (**D**, **I**), conditions as indicated. CCL2 treatment increased area of surface GluA1, an effect totally blocked by KN-93 and partially blocked by PKI14-22 (DMSO + BSA, *n =* 80; DMSO + CCL2, *n =* 88; KN + BSA, *n =* 73; KN + CCL2, *n =* 66; PKI + BSA, n= 51; PKI + CCL2, *n* = 56; significance as indicated on graph). **C**, **E** Cumulative probability distribution of area (**C**) and intensity (**E**) (for both plots, DMSO + CCL2 *vs.* all others: *P* < 0.001, no significant differences among other 3 conditions). **H** Cumulative probability distribution of area in (**G**) (DMSO + BSA *vs.* DMSO + CCL2: *P* < 0.001; DMSO + BSA *vs.* PKI + CCL2: *P* < 0.01; DMSO + CCL2 *vs.* PKI + CCL2: *P* = 0.05). **J** Cumulative probability distribution of intensity in (**I**) (DMSO + BSA *vs.* DMSO + CCL2: *P* < 0.001; DMSO + BSA *vs.* PKI + CCL2: *P* < 0.05; DMSO + CCL2 *vs.* PKI + CCL2: *P* < 0.05). Data for DMSO + BSA and DMSO + CCL2 conditions in **6G** and **6I** are the same as that in **6B** and **6D**, respectively. Each data point represents one neuron. Kolmogorov-Smirnov test for (**C**, **E**, **H**, **J**); two-way ANOVA followed by Bonferroni’s *post hoc* test for (**B**, **D**, **G**, **I**). Scale bar, 25 μm.

Gα_q_ activation elevates intracellular calcium levels and promotes activation of CaMKII through phosphorylation at Threonine 286 (T286) [79–81], while Gα_s_ and cAMP are known to activate PKA activity [82, 83]. We examined the contributions of CaMKII and PKA in mediating the effects of CCL2, by pretreating cultured hippocampal neurons with the CaMKII inhibitor KN-93 (Fig. 6A–E) or the PKA inhibitor PKI 14-22, (Fig. 6F–J). KN-93 abolished the effects of CCL2 in promoting membrane insertion of GluA1, both in terms of area (DMSO + CCL2 *vs.* all others, *P* < 0.001; no significant differences among other 3 conditions, Fig. 6B) and intensity (Fig. 6D). PKI 14-22, on the other hand, only had partial effects on GluA1 area (DMSO + BSA *vs.* DMSO + CCL2, *P* < 0.001; DMSO + BSA *vs.* PKI + CCL2, *P* < 0.01; DMSO + CCL2 *vs.* PKI + CCL2, *P* = 0.30, Fig. 6G) and intensity (DMSO + BSA *vs.* DMSO + CCL2, *P* < 0.001; DMSO + BSA *vs.* PKI + CCL2, *P* < 0.05; DMSO + CCL2 *vs.* PKI + CCL2, *P* < 0.05, Fig. 6I).

These results are inconsistent with no significant Gα_s_ signaling following CCL2 treatment. We propose possible reasons for this discrepancy in the Discussion section.

### CCL2-CCR2 Signaling Regulates GluA1 Phosphorylation

How do CaMKII and PKA regulate surface AMPA receptor levels? Previous studies showed that phosphorylation of the C-terminal domain of GluA1 at serine 845 (S845) and S831 sites, respectively the targets of CaMKII and PKA, is associated with potentiation of synaptic transmission [40–42, 45, 52]. Does CCL2 mediate its effects on excitatory synaptic transmission through these sites? To address this question, we used an *in vivo* manipulation which we previously showed to significantly increase CCL2 expression [35], namely intraperitoneal (i.p.) injection of lipopolysaccharides (LPS), an often used model for inducing neuroinflammation [84–86]. Consistent with our previous report [35], i.p. injection of LPS in postnatal day 14 (P14) mice induced high-level expression of multiple cytokines in the hippocampus, including CCL2 (Fig. S2A–D), and also increased immobility in the tail suspension test (TST) (Fig. S2G) [35].

**Fig. 7:**
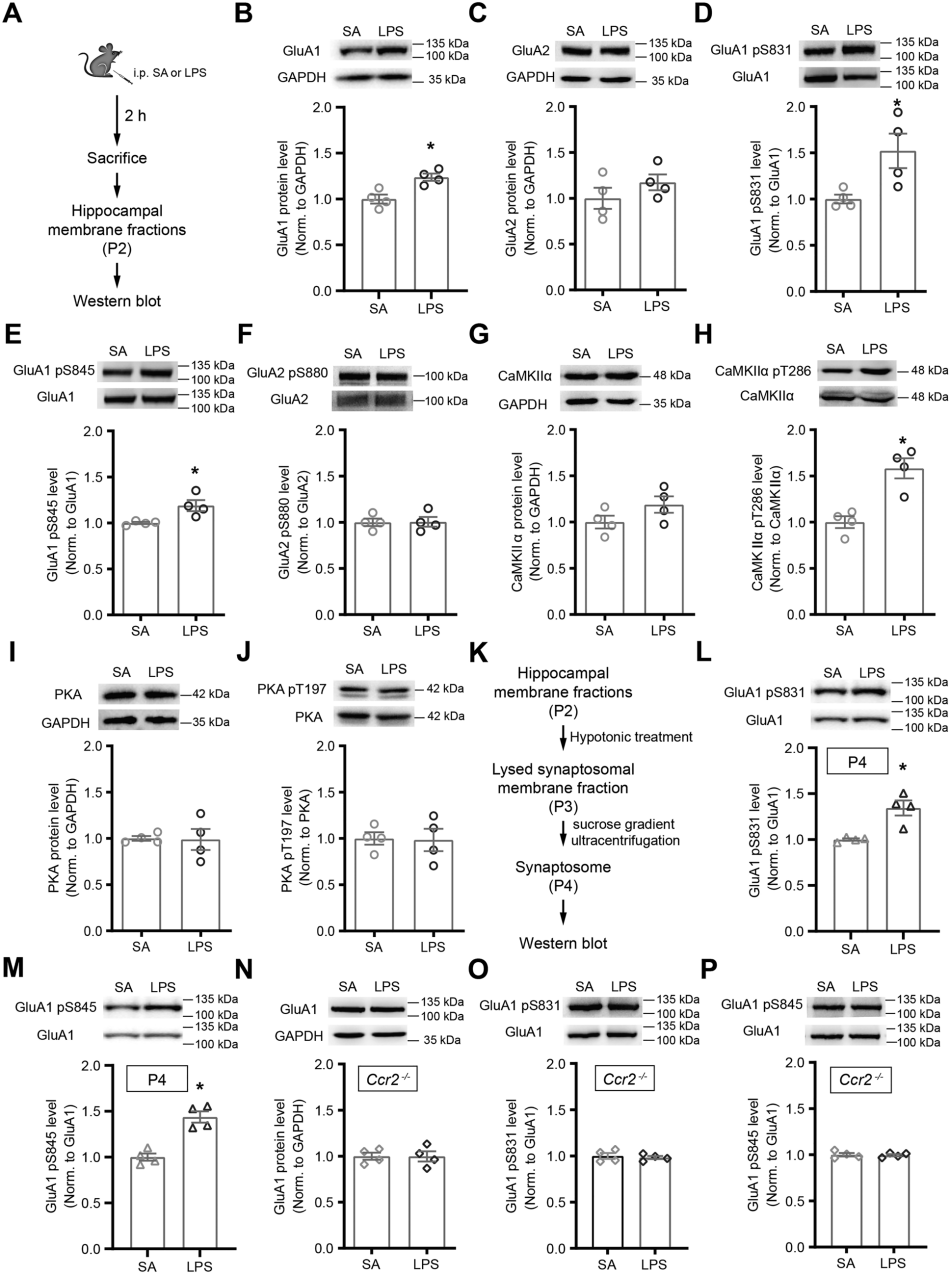
LPS-treatment elevates expression of membrane-associated GluA1 and its phosphorylation, an effect blocked in *Ccr2* knockout mice. **A** Schematic of the experimental procedure. **B**, **C** Representative immunoblots and quantitation of GluA1 and GluA2 levels from membrane fractions of hippocampi of Ctrl (SA) and LPS-treated WT mice. **D**, **E** Representative immunoblots and quantitation of S831 and S845 phosphorylation of GluA1, respectively. **F** Representative immunoblots and quantitation of GluA2 S880 phosphorylation. **G**–**J** Representative immunoblots and quantitation of CaMKIIα and PKA protein levels and their phosphorylation states from hippocampal membrane fractions of Ctrl (SA) and LPS-treated WT mice, conditions as indicated. **K** Workflow of synaptosome (P4) purification from the P2 fraction. **L**, **M** Representative immunoblots and quantitation of S831 and S845 phosphorylation of synaptosomal GluA1, respectively. **N**–**P** Representative immunoblots and quantitation of total and phosphorylated GluA1 from Ctrl and LPS-treated *Ccr2*^*−/−*^ mice. For phosphorylation measurements, immunoblots were stripped and reprobed with antibodies against total protein. Each data point represents one mouse. Mann-Whitney test.

In hippocampal samples prepared from mice 2 h after LPS treatment, the membrane-associated level of GluA1 was significantly elevated, (SA, 1.00 ± 0.05; LPS, 1.24 ± 0.04; *P* < 0.05; Fig. 7A, B), while that of GluA2, another highly expressed AMPA receptor subunit [87–89], was not altered significantly (SA, 1.00 ± 0.12; LPS, 1.18 ± 0.09; *P* = 0.34; Fig. 7C). No obvious changes in the mRNA level of these proteins were observed (Fig. S2E, F), suggesting that LPS-dependent upregulation of GluA1 occurred at the protein, but not the mRNA, level.

In LPS-treated mice, phosphorylation levels of both S831 (SA, 1.00 ± 0.05; LPS, 1.52 ± 0.19; *P* < 0.05; Fig. 7D) and S845 (SA, 1.00 ± 0.01; LPS, 1.19 ± 0.06; *P* < 0.05; Fig. 7E), ratioed to total GluA1 in membrane-associated fractions, were significantly elevated, as compared to littermates injected with saline. Consistent with no significant changes in GluA2 level, phosphorylation of its S880 site [90] was not affected (SA, 1.00 ± 0.04; LPS, 1.01 ± 0.05; *P* = 0.89; Fig. 7F).

Consistent with CCL2 regulating S831 phosphorylation (Fig. 7D) of GluA1 via CaMKII signaling, LPS treatment significantly increased T286 phosphorylation of CaMKII (SA, 1.00 ± 0.06; LPS, 1.58 ± 0.11; *P* < 0.05; Fig. 7H), without affecting total CaMKII protein level (SA, 1.00 ± 0.07; LPS, 1.19 ± 0.09; *P* = 0.20; Fig. 7G). Neither the protein level of PKA (SA, 1.00 ± 0.03; LPS, 0.99 ± 0.11; *P* = 0.89; Fig. 7I) nor its phosphorylation level at the Threonine 197 (T197) site [91, 92] (SA, 1.00 ± 0.07; LPS, 0.98 ± 0.12; *P* > 0.99; Fig. 7J), was affected by LPS treatment.

To further determine whether changes in the phosphorylation level of S831 and S845 really occurred at synapses, we purified synaptosomes using standard protocols (Fig. 7K) and confirmed that LPS-treatment significantly upregulated phosphorylation level of synaptic GluA1 at both S831 (SA, 1.00 ± 0.01; LPS, 1.34 ± 0.08; *P* < 0.05; Fig. 7L) and S845 (SA, 1.00 ± 0.04; LPS, 1.44 ± 0.06; *P* < 0.05; Fig. 7M) sites.

The effects of LPS-induced upregulation of membrane-associated GluA1 (SA, 1.00 ± 0.04; LPS, 1.00 ± 0.06; *P* > 0.99; Fig. 7N), as well as levels of S831 (SA, 1.00 ± 0.03; LPS, 0.98 ± 0.02; *P* = 0.89; Fig. 7O) and S845 (SA, 1.00 ± 0.02; LPS, 1.00 ± 0.01; *P* = 0.89; Fig. 7P), were effectively blocked in *Ccr2* knockout mice, demonstrating that CCL2-CCR2 signaling is the main signaling pathway mediating LPS-induced upregulation of membrane-associated GluA1 level.

## Discussion

### Mechanism Mediating CCL2-dependent Regulation of Surface GluA1 Trafficking

**Fig. 8:**
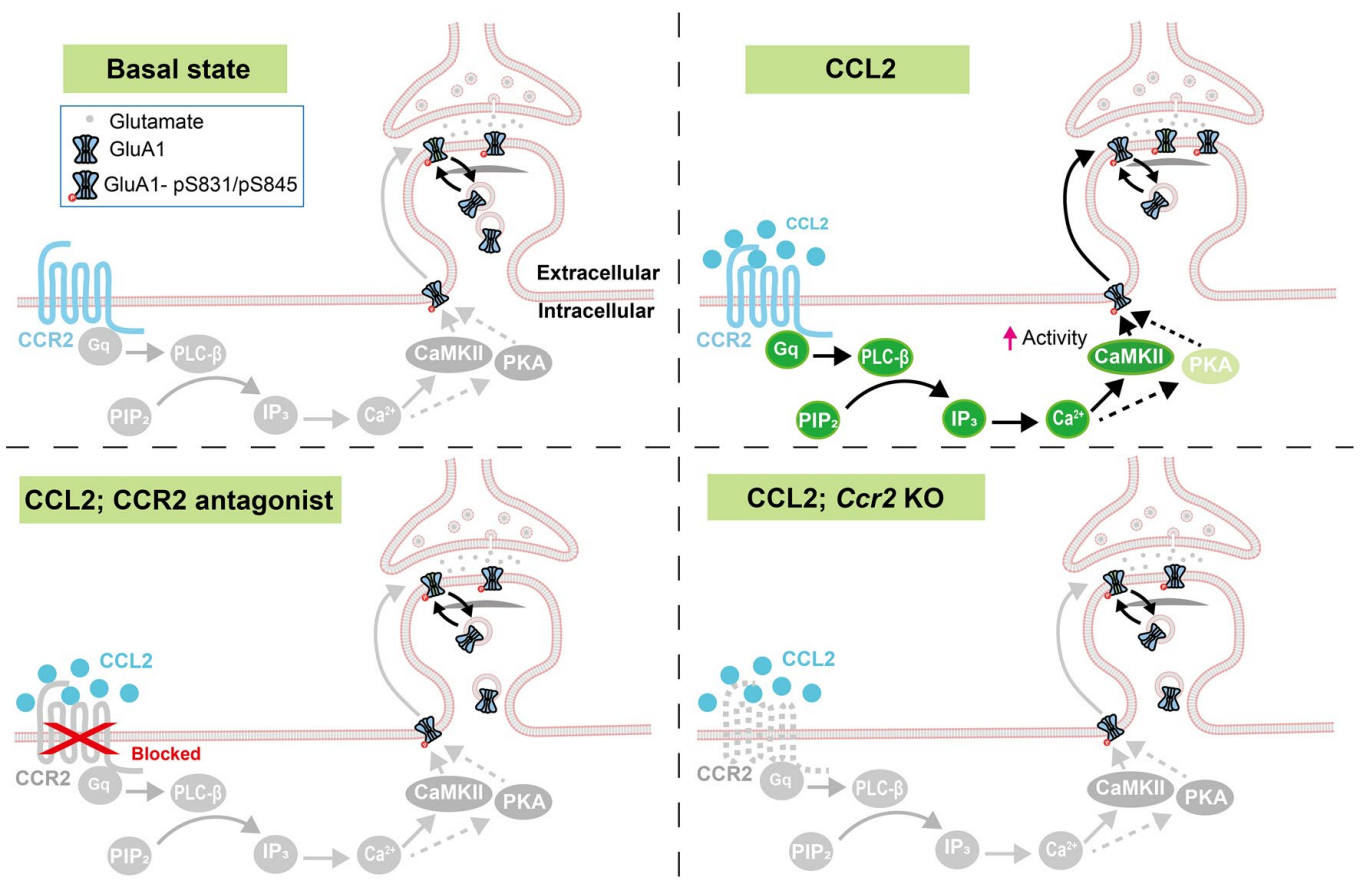
A proposed model for CCR2-mediated GluA1 surface trafficking in hippocampal neurons under the inflammatory state. Under the basal state, due to low levels of CCL2, CCR2, and its downstream signaling do not contribute significantly to the regulation of surface GluA1 level (upper left). During neuroinflammation, the CCL2 level is significantly elevated, activating CCR2, and promoting GluA1 membrane expression mainly via Gα_q_, Ca^2+^, and CaMKII signaling, with contribution from PKA signaling (upper right). When CCR2 is blocked (lower left) or deficient (lower right), this pathway is not activated, resulting in failure to deliver GluA1 to the surface.

Using a combination of immunocytochemistry, live imaging, and whole-cell patch clamp recordings, we showed that CCL2 primarily signals through CCR2, Gα_q_, calcium, and CaMKII to regulate the surface expression of GluA1, in cultured hippocampal neurons. Consistently, i.p. injection of LPS increased phosphorylation levels of CaMKII at the T286 site and of GluA1 at the S831 site. Based on the above results, we propose this to be the main pathway through which CCL2 regulates surface AMPA receptor expression and excitatory synaptic transmission (Fig. 8).

Does the alternative Gα_s_ pathway also contribute? Here, there were some inconsistencies, LPS-induced upregulation of GluA1 S845 phosphorylation was blocked in *Ccr2* knockout mice, but LPS injection *in vivo* or CCL2 application *in vitro* did not alter Gα_s_ activity, PKA level or T197 phosphorylation of PKA, while application of the PKA inhibitor PKI 14-22 only partially blocked CCL2-induced upregulation of surface GluA1 level. These discrepancies could be due to one of several reasons. A recent study suggested that Gα_q_-coupled GPCR activation may increase PKA activity via calcium or protein kinase C (PKC)-dependent pathways [93]. If the effects on PKA were transient, we may have captured increased GluA1 S845 phosphorylation without observing a significant increase in PKA activity or phosphorylation level. Given that GluA1 phosphorylation at S845 constitutes a relatively small fraction of total GluA1 [41, 94], little PKA activity would be needed to induce significant changes in phosphorylation at this site. Consistently, the PKA inhibitor at least partially inhibited CCL2-induced surface GluA1 expression. We also cannot exclude the contribution of Gα_i_, which opposes Gα_s_ and may mask its activity, as we observed Gα_i_ activity increase following CCL2 application in HEK cells (data not shown).

Putting all results together, we propose the main pathway activated by CCL2 in hippocampal neurons to be mediated through CCR2, Gα_q_, Ca^2+^, CaMKII, and GluA1 signaling. Given that we observed increased surface expression of GluA1, S845 phosphorylation-dependent surface AMPA receptor trafficking likely contributes. Possible mechanisms include PKA-dependent phosphorylation of S845, cooperativity between the S845 and S831 sites [95], and/or interactions with auxiliary proteins in the postsynaptic density (PSD) [52].

### Implications for Regulation of Synaptic Plasticity by Chemokines

Is the above-identified pathway specific for the hippocampus or is it more general? In a previous study, we showed that the application of CCL2 onto acute hippocampal slices increased excitatory synaptic transmission in CA1 and CA3 hippocampal pyramidal neurons, as well as L2/3 pyramidal neurons of the primary somatosensory cortex, and granule cells of the dentate gyrus [35]. Similar effects were observed in spinal cord lamina II neurons [37], and LPS-activated neurons in the ventral-medial preoptic area (VMPO) [69]. Thus, the signaling mechanism that we have identified is likely common to multiple neuron types.

What about chemokine signaling more generally? Chemokine receptors belong to the GPCR family. In addition to CCL2, CCL5, CX3CL1, and CXCL12 have also been reported to regulate synaptic transmission and plasticity [19–22]. These are only a small fraction of known chemokines. The complementary *in vivo* and *in vitro* methods used in this study provide a systematic approach to investigating the effects of chemokines on regulating synaptic transmission. Given the emerging roles of chemokines and cytokines as important communicators between the nervous and immune systems, and as regulators of neural function [19–22, 96], a systematic analysis of their mechanisms of action is critical to a better understanding of their physiological functions.

## Supplementary Information

Below is the link to the electronic supplementary material.

Supplementary file1 (PDF 2136 KB)

## Data Availability

The datasets used and/or analyzed in the current study are available from the lead contact on reasonable request.
