# Supplementary material for: The Chemokine CCL2 Promotes Excitatory Synaptic Transmission in Hippocampal Neurons via GluA1 Subunit Trafficking
Source: Neurosci Bull. 2024 Jul 2;40(11):1649–66. doi: 10.1007/s12264-024-01236-9 (PMC11607194; doi:10.1007/s12264-024-01236-9)
Supplement: Supplementary file 1 — Supplementary file1 (PDF 2136 KB) [file 12264_2024_1236_MOESM1_ESM.pdf]

Supplementary Figures and Legends

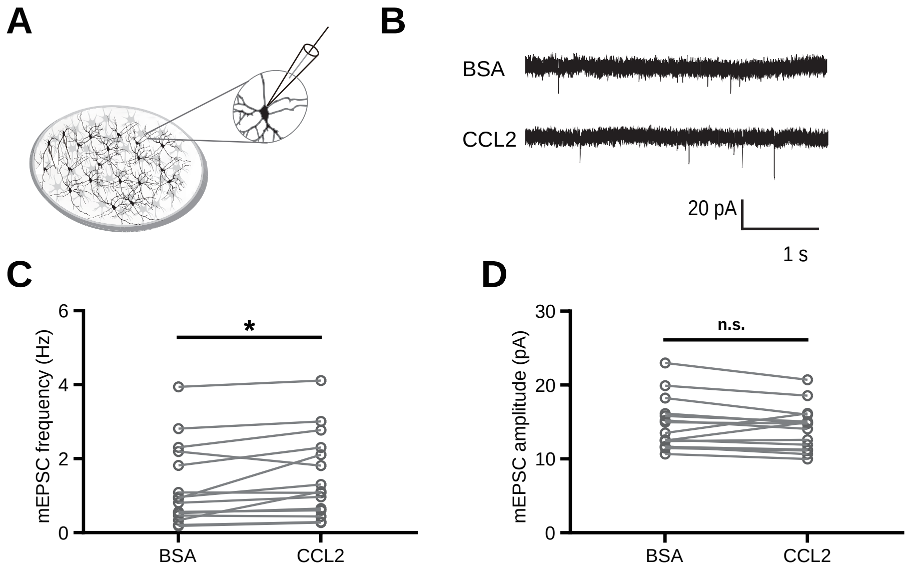

Figure S1. CCL2 application increases mEPSC frequency in cultured hippocampal neurons.

A Schematic of experimental procedure. **B-D** Representative traces (**B**) and summary data (**C**, **D**) of the effects of CCL2 on mEPSC frequency and amplitude (Frequency: BSA, 1.28 ± 0.29 Hz; CCL2, 1.52 ± 0.29 Hz, $P < 0.05$; Amplitude: BSA, 14.65 ± 0.91 pA; CCL2, 14.18 ± 0.78 pA, $P = 0.22$; n = 15 for both conditions). Recordings are made 5 to 10 min after BSA or CCL2 application. Each data point represents one neuron. Paired two-tailed *t*-test.

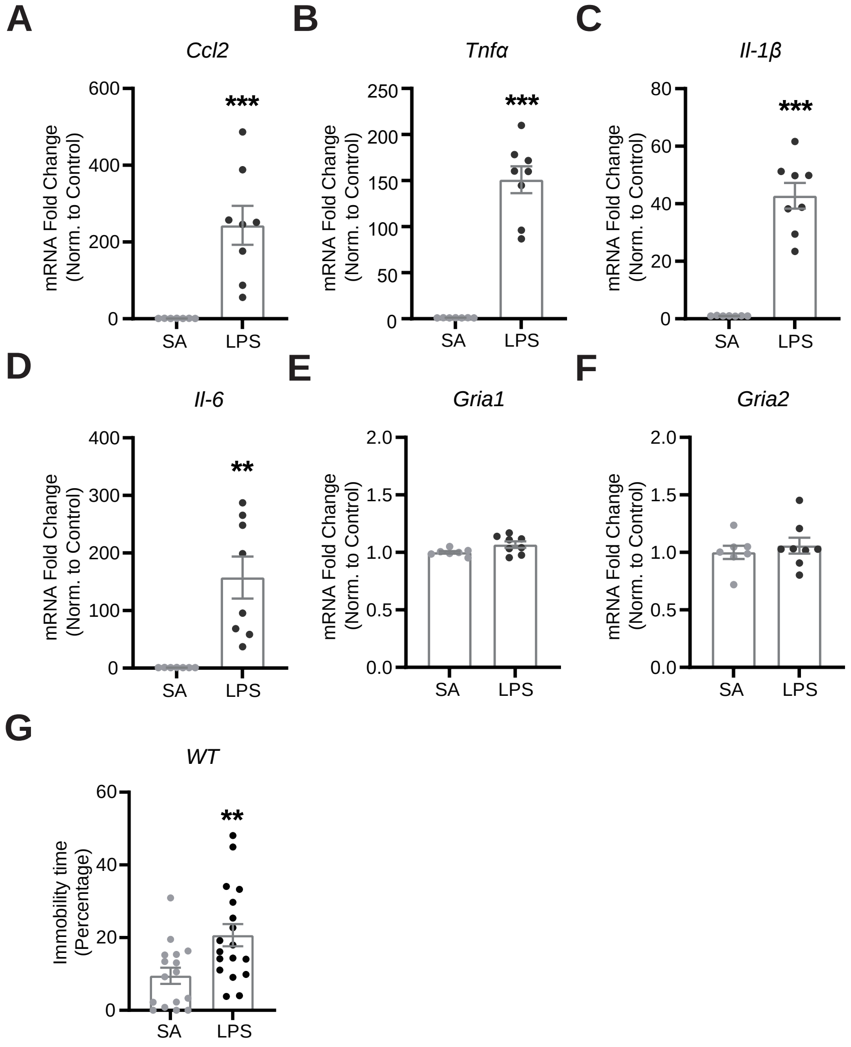

Figure S2. mRNA expression of cytokines and AMPA receptor subunits in the hippocampus following LPS treatment.

A-D Fold change in mRNA level of various cytokines in the hippocampus of P14 mice2 h after i.p. injection of saline (SA) or LPS (SA, n = 7; LPS, n = 8; A: $P < 0.01$; B: $P < 0.001$; C: $P < 0.001$; D: $P < 0.01$). **E**, **F** Fold change in mRNA level of GluA1 and GluA2 in the hippocampus of P14 mice 2 h after i.p. injection of saline (SA) or LPS (E: $P = 0.05$; F: $P = 0.53$). **G** Percentage immobility time in tail-suspension test of mice 2 h after i.p. injection of saline (SA) or LPS (SA, n = 16; LPS, n = 18; A: $P < 0.01$). Each data point represents one mouse. Unpaired *t*-test.
